# The ratio of phosphatidylcholines to lysophosphatidylcholines in plasma differentiates healthy controls from patients with Alzheimer's disease and mild cognitive impairment

**DOI:** 10.1016/j.dadm.2015.05.003

**Published:** 2015-06-28

**Authors:** Kristaps Klavins, Therese Koal, Guido Dallmann, Josef Marksteiner, Georg Kemmler, Christian Humpel

**Affiliations:** a Biocrates Life Sciences AG, Innsbruck, Austria; b State Psychiatric Hospital, Hall in Tirol, Austria; c Department of Psychiatry and Psychotherapy, University Clinic of General and Social Psychiatry, Medical University of Innsbruck, Innsbruck, Austria

**Keywords:** Alzheimer's disease, Mild cognitive impairment, Diagnosis, Plasma, Metabolomics

## Abstract

**Background:**

Metabolomic processes have been identified as being strongly linked to the development of Alzheimer's disease (AD). Thus, lipid metabolites appear to be highly useful as diagnostic substrates for the diagnosis of AD and mild cognitive impairment (MCI) in plasma.

**Methods:**

We analyzed plasma samples from controls (n = 35), MCI (n = 33), and AD patients (n = 43) using the AbsoluteIDQ p180 Kit (Biocrates Life Sciences), which included quantitative analysis of 40 acylcarnitines, 21 amino acids, 19 biogenic amines, 15 sphingolipids, 90 glycerophospholipids, and sum of hexoses.

**Results:**

We found that individual lipid metabolites can differentiate controls from MCI and AD with relevant significance. However, the ratio between PC aa C34:4 and lysoPC a C18:2 differentiates controls from MCI (*P* = .0000007) and from AD (*P* = .0000009) with greater significance.

**Conclusions:**

The results provide evidence that the ratio of these two lipid metabolites is useful for diagnosing MCI and AD with an accuracy of 82%–85%.

## 1 Introduction

Alzheimer's disease (AD) is a progressive neurodegenerative disease of the brain characterized by extracellular β-amyloid plaques, intraneuronal neurofibrillary tau tangles, inflammation and glial responses, vascular dysfunction, cholinergic neurodegeneration, and synapse loss that directly correlates with cognitive decline and memory loss. The causes of AD are not known but the most prominent hypothesis is the β-amyloid cascade hypothesis [1]. However, dysfunctions in tau phosphorylation may also play a role independent of β-amyloid [2] but recent evidence indicates that both proteins interact [3]. More and more clinical and basic data show that a vascular risk factors may play a role in the development of AD [4] and that a dysfunction of the blood-brain barrier may also account for dysregulated clearing of β-amyloid from the brain [5].

The diagnosis of possible or probable AD is made on the basis of a time-consuming psychological test and clinical examination by excluding other psychiatric and neurologic diseases. Brain imaging and the analysis of cerebrospinal fluid (CSF) samples are important but expensive tools for verifying the diagnosis. Due to the invasive nature of CSF collection, blood biomarkers need to be found to allow screening and multiple analyses of patients, especially those with mild cognitive impairment (MCI). There is more and more evidence to show that a single biomarker cannot yield enough sensitivity and specificity to diagnose AD [6], [7]. Thus, multiple analyses and the generation of a patient-specific signature are state-of-the-art.

In 2007, Ray et al. [8] claimed to diagnose AD from plasma using a panel of 18 biomarkers. However, several groups including ours failed to reproduce this finding [9], [10]. Recently, Mapstone et al. [11] demonstrated that a set of 10 endogenous lipids from peripheral blood can predict phenoconversion to either amnestic MCI or AD within a 2–3 year time frame with over 90% accuracy. In fact, there are clear indications that metabolic processes are linked to the development and pathology of AD [12], [13] and metabolomics is turning out to be a novel fascinating method for analyzing a large panel of lipid metabolites [14]. Two recent articles clearly demonstrate that plasma lipidomics is associated with AD [15] and that a blood-based 7-metabolite signature may diagnose early AD [16].

The aim of the present study was to analyze the metabolome in plasma samples of controls, MCI, and AD patients. We quantitatively analyzed 40 acylcarnitine metabolites, 21 amino acids, 19 biogenic amines, 15 sphingolipids, and 90 glycerophospholipids using the AbsoluteIDQ p180 Kit (Biocrates Life Sciences AG, Innsbruck, Austria). We here show that several lipids are altered in MCI and AD EDTA plasma and that two lipids or their ratio provide a potent biomarker for distinguishing MCI and AD from controls.

## 2 Methods

### 2.1 Patients

A total of 111 samples (healthy controls, AD, and MCI) were included in this study during the sample collection period 2004–2012. All patients were >70 years and were recruited from the memory clinics at the Department of Psychiatry of Innsbruck Medical University and Hall in Tirol State Hospital, both in Austria. Healthy subjects, mainly healthy caregivers and volunteers without any cognitive impairment, were also recruited at these sites. Psychiatrists clinically examined all subjects, performed a standardized psychiatric and neurologic examination, reviewed medical records, and all subjects underwent a neuropsychological assessment (mini mental state examination [MMSE] and geriatric depression scale [GDS]). Exclusion criteria for healthy subjects and patients suffering from MCI or AD included other psychiatric or neurologic diseases or diseases including cancer, vascular diseases, or other diseases with clinically significant hepatic, renal, pulmonary, metabolic or endocrine disturbances, and inflammation. Participants underwent continuous statin or ezetimibe treatment for at least 3 months before study entry. No patient had a cholesterol level >240 mg/dL that was not treated with a statin or ezetimibe. The procedure for diagnosis has been described by us in detail elsewhere [9], [17]. The study was approved by the Local Ethics Committee of Innsbruck Medical University and was performed in accordance with the Helsinki Declaration. All subjects gave written informed consent.

### 2.2 Blood collection

Blood samples were taken between 9:30 and 11:00 AM. Participants had a fasting time ranging from 1 to 3 hours. Breakfast foods taken by the participants were not noted. After a patient was assigned to a group, 10 mL of EDTA blood was collected and processed. The samples were centrifuged (400 × *g*, 30 min), and the upper plasma phase was immediately frozen at −80°C. Blood processing time was 4.3 ± 0.2 hours; the blood from 26% of the patients was processed the next day. Thus, mean processing time was 10.3 ± 1.8 hours (controls), 8.8 ± 1.4 hours (MCI), and 10.3 ± 1.5 hours (AD). Processing times did not differ between groups. To test the stability of the metabolites, EDTA blood was taken from non–cognitively impaired volunteers and AD patients and processed immediately (t = 0) or it was left at room temperature for 1, 2, or 3 days and then processed. To test stability over 2 years at −20°C, blood was taken from a volunteer, processed and analyzed immediately or stored at −20°C for 2 years, and then analyzed.

### 2.3 Metabolomic analysis

The endogenous metabolites were analyzed with a targeted quantitative and quality controlled metabolomics approach using the AbsoluteIDQ p180 Kit (Biocrates Life Science AG) as described recently by us [18]. This validated assay allows the comprehensive identification and the quantification of 186 endogenous metabolites including 21 amino acids, 19 biogenic amine, 40 acylcarnitines, 76 phosphatidylcholines (PCs), 14 lysophosphatidylcholines (lysoPCs), 15 sphingomyelins, and sum of hexoses. Analyzed glycerophospholipids are differentiated according to the presence of ester and ether bonds in the glycerol moiety. The “aa” indicates that fatty acids are at the sn-1 and the sn-2 position bound to the glycerol backbone via ester bonds, whereas “ae” denotes that fatty acid at the sn-1 position is bound via ether bond. Total number of carbon atoms and double bonds present in lipid fatty acid chains are denoted as “C x:y,” where x indicates the number of carbons and y the number of double bonds. Sample preparation was performed according to the user manual. Samples were randomized, and multiple quality control samples were included in the measurement sequence. Intra-assay variation was 3.8 ± 0.8% (n = 32) and inter-assay variation 4.4 ± 1.1% (n = 32).

### 2.4 Statistical analysis

Plasma metabolites were checked for deviations from a normal distribution using the Shapiro-Wilk test [19]. Metabolites with a nonnormal distribution were log-transformed before analysis. One-way analysis of variance was used to compare the three diagnostic groups (healthy controls, MCI, and AD) with respect to the plasma levels. Post hoc pairwise comparisons were performed with Fisher's least-significant difference method. In the case of the three groups, this method provides valid *P* values without correction for multiple testing [20]. For all markers, significance was adjusted for multiple testing using the Bonferroni method, dividing the usual significance level (α = 0.05) by the number of tests performed (α_corrected_ = 0.05/183 = 0.0002732). Performance of each metabolite and metabolite ratios as potential biomarker was evaluated with receiver operating characteristic (ROC) analysis. All lipid metabolites including the ratios were entered in a ROC analysis to obtain estimates of the sensitivity and specificity and of the area under the ROC curve (AUC). Optimal cutoff levels were determined such that the sum of sensitivity and specificity was maximized. Bias-corrected estimates of sensitivity and specificity were obtained using leave-one-out cross-validation [21]. Calculation of 95% confidence intervals for cutoff levels was based on confidence limits for the ED50 as provided in the probit regression routine in SPSS, version 22. Moreover, positive and negative predictive values were calculated based on the prevalence of MCI and AD as given in the sample investigated (this was representative for the two participating memory clinics). The Fagan nomogram was used to provide a graphical presentation of pretest and posttest probabilities for MCI or AD [22]; (http://araw.mede.uic.edu/cgi-bin/testcalc.pl).

## 3 Results

### 3.1 Screening of metabolites

The present study investigated 43 AD, 33 MCI, and 35 control plasma samples with a targeted quantitative metabolomics approach (Supplementary Table 1). MCI patients did not differ in age, gender, or GDS but had slightly reduced MMSE scores as compared with those of controls (Table 1). AD patients were slightly older (*P* < .05) and had highly significantly reduced MMSE scores (Table 1). Statistical analysis identified significant alteration in glycerophospholipid levels. The levels of five phosphatidylcholines (PC aa C34:4, C36:6, C38:3, C40:5, and C40:6) were lower and levels of two lysoPCs (lysoPC a C18:1 and lysoPC a C18:2) were higher in MCI and AD plasma samples than in controls (Table 2). The metabolites with best performance for differentiating AD and MCI patients from controls were PC aa C34:4 (AUC 0.76) and PC aa C40:5 (AUC 0.77). Adjustment for age by analysis of covariance had little effect on the results and left the *P* values in Table 2 almost unchanged. Two plasma amino acids were altered in AD as compared with those of controls (glycine *P* = .017 and valine *P* = .059) but these data should be considered with care because amino acids are markedly altered by fasting (see Discussion in the following).

### 3.2 Ratio of lipid metabolites

**Fig. 1:**
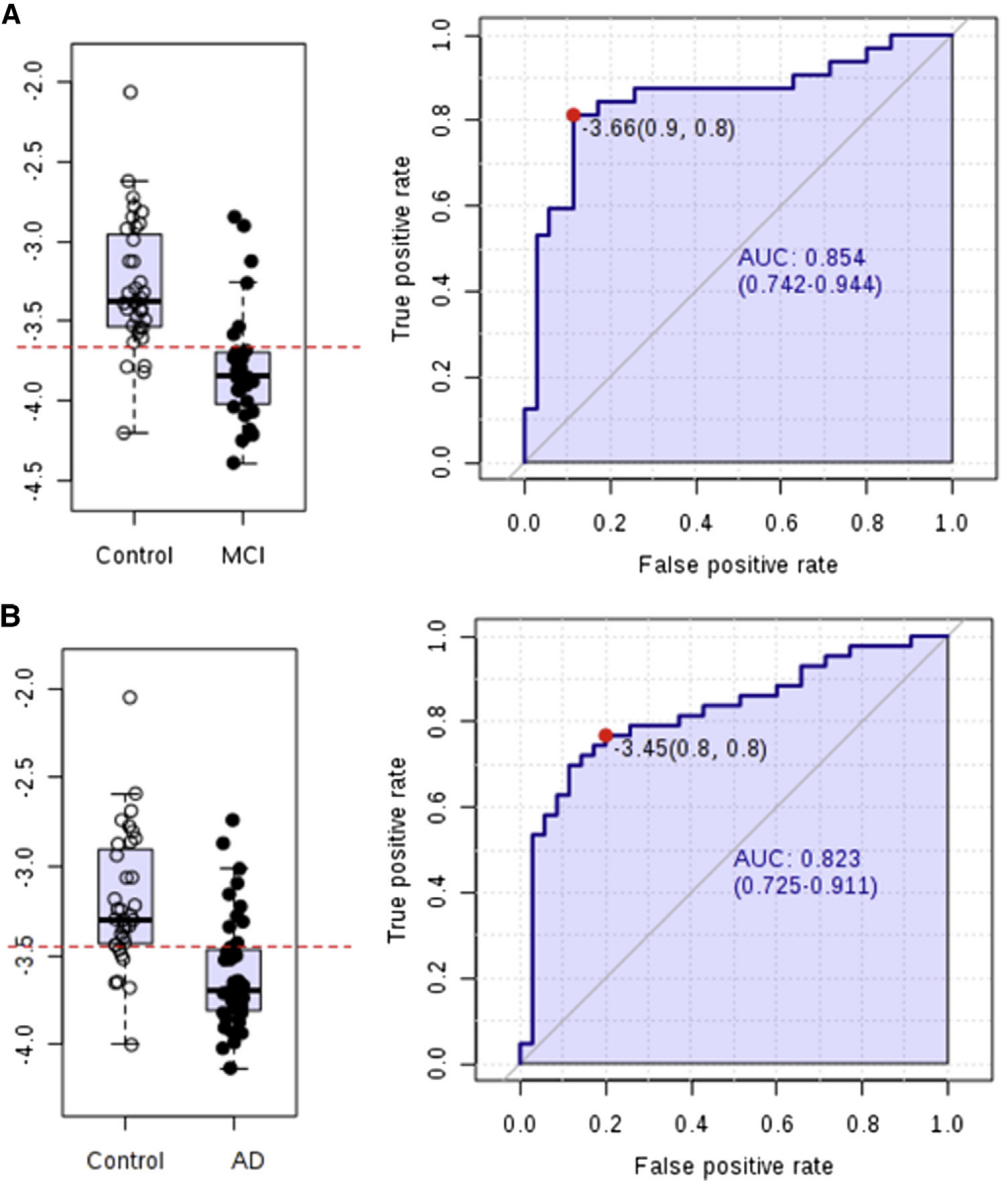
Raw values and ROC curves of the metabolite ratio with the highest statistical significance in MCI (A) and AD (B) patients as compared with those of controls. Abbreviations: MCI, mild cognitive impairment; AUC, area under the ROC curve; AD, Alzheimer's disease; ROC, receiver operating characteristic.

**Fig. 2:**
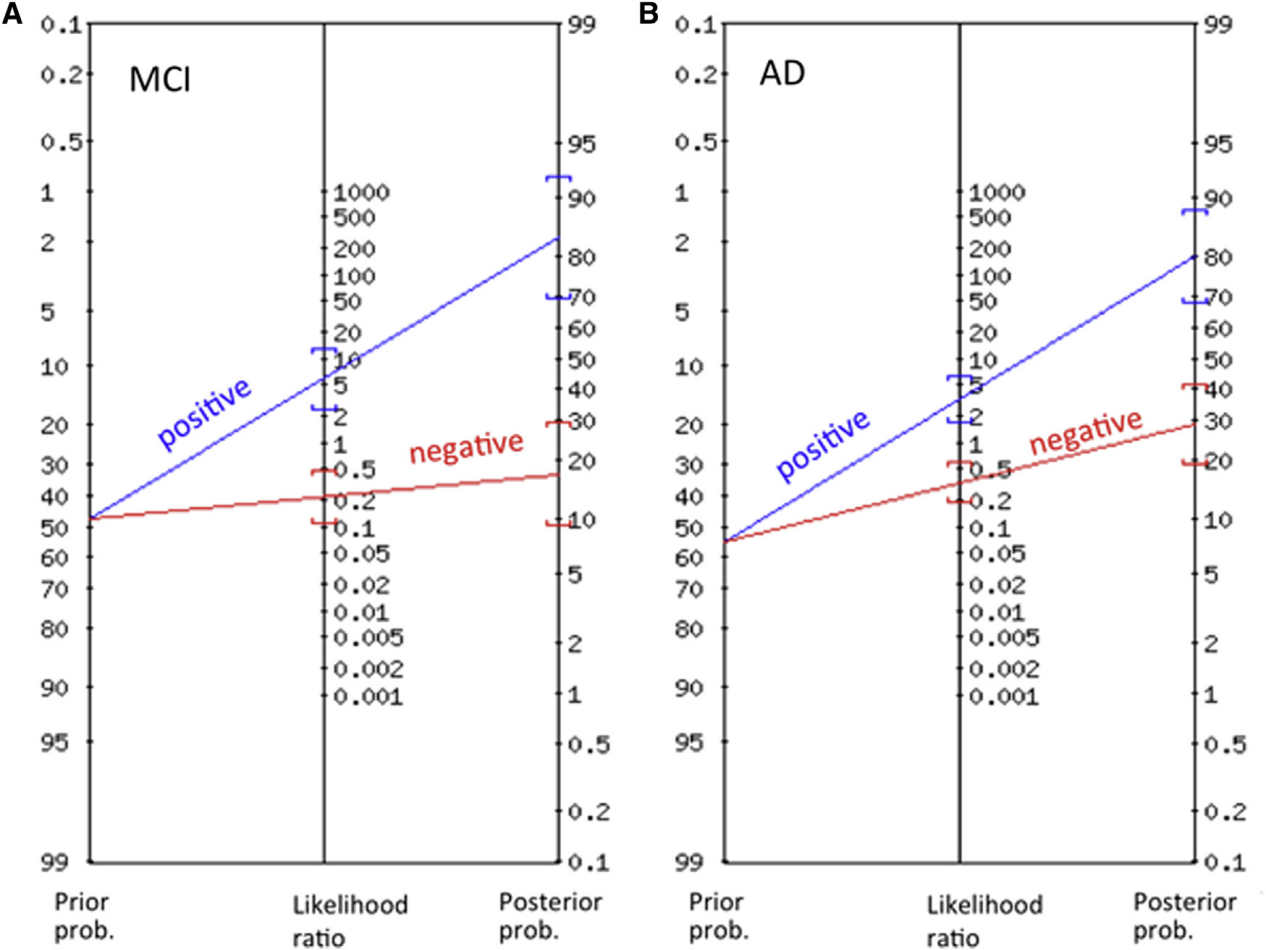
Fagan nomogram showing pretest and posttest probabilities of developing MCI (A) or AD (B) (blue line: subjects with positive test result, red line: subjects with negative test result). Abbreviations: MCI, mild cognitive impairment; AD, Alzheimer's disease.

When the ratios between PC aa C34:4 or C36:5 or C36:6 and lysoPC a C18:1 or C18:2 were calculated, the significance to differentiate controls from MCI and AD patients was dramatically higher (Table 2). Our data show that the ratio between PC aa C34:4 and lysoPC a C18:2 highly significantly differentiates controls from MCI patients (*P* = .0000007; AUC under ROC = 0.85) and controls from AD patients (*P* = .0000009; AUC under ROC = 0.82; Table 2; Fig. 1). More detailed analyses reveal that the mentioned ratio shows good performance in terms of sensitivity, specificity, and both positive and negative predictive value for differentiating MCI patients from controls (all values >0.8) and satisfactory accuracy for distinguishing AD patients from controls (all values >0.7; Table 3). A graphical presentation of pretest and posttest probabilities by Fagan nomogram of MCI and AD is given in Fig. 2. The ratios between PC aa C34:4/lysoPC a C18:1 and PC aa C36:6/lysoPC a C18:1 and PC aa C36:6/lysoPC a C18:2 were similar but not as potent. In no case did the ratios differentiate MCI from AD patients (Table 2).

### 3.3 Stability of the lipids

To test the lipid stability during the processing of blood samples, we analyzed our two best lipids in blood from a cognitively nonimpaired volunteer (Table 4A) and a severe AD patient (Table 4B). Our data show that the plasma levels of lysoPC a C18:2 were significantly higher in blood samples after storage for 24 hours at room temperature, whereas plasma levels of PC aa C34:4 were significantly lower in blood samples after storage for 48 hours at room temperature. However, more importantly, the ratio between these two lipids was significantly lower after storage of blood samples for 24 hours at room temperature. Thus, these data clearly demonstrate the instability of both lipids when blood samples are stored for a prolonged time at room temperature. Consequently, it was important to test the stability of these two metabolites in frozen samples. Our results from long-term stability (Table 4C) show that lipids are stable at −20°C for up to 2 years. Thus, for further validation studies, samples should be processed and frozen as soon as possible after sampling. Otherwise, metabolite instability might cause a false-negative diagnosis.

## 4 Discussion

In the present study, we screened 186 metabolites in plasma from control, MCI, and AD patients. Our data show that two lipids significantly differentiate MCI and AD patients from healthy controls, and the ratio is even more significant and may provide a novel biomarker. Both lipids display instability in whole blood stored for 24 hours at room temperature but are stable in frozen plasma for up to 2 years.

A decrease in PCs and lysoPCs in patients with AD has been reported before in peripheral blood samples [23], [24], [25], postmortem brain samples [26], and animal models [26], [27]. Mapstone et al. [11] demonstrated that a set of 10 lipids (C3, lysoPC a C18:2, PC aa C36:6, C16:1- OH, PC aa C38:0, PC aa C38:6, PC aa C40:1, PC aa C40:2, PC aa C40:6, and PC ae C40:6) from peripheral blood can be used to predict phenoconversion from control to either MCI or AD with over 90% accuracy. All these metabolites were also measured with the same technological platform in our sample set. As a matter of fact, three of them, PC aa C34:4, PC aa C38:3 and PC aa C40:5, retained significant after post-Bonferroni correction for multiple testing. This underlines the relevance of PCs in AD pathophysiology as previously indicated [11], [15]. The reduced levels of PCs might be linked with aberrant activity of phospholipase A_2_ (PLA_2_). PLA_2_ are enzymes that catalyse cleavage of fatty acids from the sn-2 position of phospholipids, producing free fatty acids and lysoPCs. It has been reported that β-amyloid_42_ peptides (that aggregate in the AD brain) increase PLA_2_ activity [28]. In fact, Hicks et al. [28] demonstrated that PLA_2_ is involved in the mechanism underlying the effect of β-amyloid_42_ oligomers on cell membrane phase properties. PLA_2_ implications in AD have been described comprehensively elsewhere [29].

Some studies also reported altered lysoPC levels in AD patient plasma [25], [30], CSF [31], and in total postmortem AD brains [26]. LysoPCs are the product of PLA_2_ catalysed reaction and are believed to be rapidly acylated with acetyl-CoA to maintain normal neural membrane composition. We hypothesize that aberrant PLA_2_ activity could also be the cause of altered levels of lysoPC, caused by a decrease in PC to lysoPC ratios in MCI and AD patients. Indeed, an increase in lysoPC levels has been observed in transgenic APP/tau mice [26], [27]. Also, several lysoPC species (including lysoPC a C18:1 and lysoPC a C18:2) have been reported to be increased in frontal cortex of postmortem AD brains [26]. Nevertheless, it has been recognized that lysoPCs are not only glycerophospholipid metabolism intermediates but also serve as mediators in multiple neuronal pathways [32]. Moreover, it has been suggested that several plasma lysoPC species are inhibitors of secreted PLA_2_ enzyme activity [33].

This study entails several limitations: (1) A main limitation of the present study is the small size of the samples. This study should therefore be followed up by large-scale multicenter studies that should also include other types of dementia, especially vascular dementia or frontotemporal lobe dementia. (2) Another limitation is that the patients were not followed up to determine the significance of prognosis of conversion to MCI or AD, as shown in the Mapstone work [11]. (3) There was a small but significant difference in age between AD patients and patients with MCI and healthy subjects. However, when adjusting for age by analyzing covariance, *P* values for differences between the three groups remained virtually unchanged. Nevertheless, further data with age-matched groups are necessary to ensure that age is not a confounder responsible for the differences between control subjects and AD patients [34]. (4) In this study, we used EDTA as an anticoagulant. It would be interesting to test if there are differences in serum, EDTA plasma, citrate plasma, or heparin plasma [35], [36]. (5) We clearly show that the lipids were not stable in whole blood samples stored at room temperature for 24 hours. Thus, future studies will have to have a processing time of 3–4 hours. Our data clearly show that prolonged storage of blood causes false-negative results. Thus, a detailed examination of all metabolites recommended for diagnosis is essential [37]. (6) It has been recognized that nutritional status can influence blood levels of several metabolite classes, especially amino acids and acylcarnitines [38], and should be considered when evaluating metabolomics data. Therefore, some authors recommend fasting before blood collection. However, to our best knowledge, the most important metabolites reported in this study are not significantly influenced by fasting or non-fasting [38]. Nevertheless, more detailed investigation will be necessary in further studies. (7) The ethnicity of patients may have an effect on metabolism; the present study used samples from a group of persons of the same ethnicity. (8) Preanalytical variations may have a significant impact on blood metabolome [39], [40]. Therefore, it is of utmost importance to consider these variances as they might cause misleading data interpretations. All our assays were analyzed under well-controlled conditions with an intra- and inter-assay variation of <5%. Taking into account all previously described limitations, it is highly recommended that blood collection and processing be standardized in future studies. A very recent consensus article from the Alzheimer's biomarkers standardization initiative [41] describes in full detail recommended parameters for future standardization.

Taken together and in conclusion, we show that different PC and lysoPC are altered in plasma of AD and MCI patients as compared with that of healthy controls. Our data suggest that the ratio of PC aa C34:4 to lysoPC a C18:2, representing the pathophysiological changes of PCs, might be highly useful as a novel plasma biomarker for the diagnosis of early dementia. The biomarker analysis in blood samples using a targeted metabolomics approach is quantitative, quick, easy, and less expensive than other assays. Further longitudinal studies and reproduction by at least two other laboratories worldwide will be necessary to introduce PC- and lysoPC-based metabolomic markers into clinical routine.

**Research in context**

1. Systematic review: We searched PubMed and Scopus to identify research studies that investigate metabolome changes in Alzheimer's disease (AD). The relevant citations are appropriately cited. References cited in the identified studies were also consulted.
2. Interpretation: The present results show significant changes in ratio of phosphatidylcholines to lysophosphatidylcholines in mild cognitive impairment and AD patients that might be potentially used for diagnosis with an accuracy of 82%–85%.
3. Future directions: Future longitudinal studies with a larger cohort need to be carried out to validate our findings. Blood collection and processing should be standardized.

## Figures and Tables

**Table 1:** Participant characteristics

Characteristics	Controls	MCI	AD
n	35	33	43
Male (%)	37.1	45.4	32.5
Age (y)	77 ± 7	75 ± 7 (NS)	81 ± 5^∗^
Blood processing (h)	10.3 ± 1.8	8.8 ± 1.4 (NS)	10.3 ± 1.5 (NS)
MMSE	29.0 ± 1.2	27.3 ± 1.2^∗^	20.6 ± 4.4^∗∗∗^
GDS	6.8 ± 6.7	5.6 ± 5.3 (NS)	7.4 ± 5.7 (NS)

Abbreviations: MCI, mild cognitive impairment; AD, Alzheimer's disease; NS, not significant; MMSE, mini mental state examination; GDS, geriatric depression scale; SD, standard deviation; ANOVA, analysis of variance.

NOTE. The Table gives the demographic data of patients who participated in this study. Values are given as mean ± SD. Statistical analysis was performed by one-way ANOVA with a subsequent Dunnett post hoc test (^∗^*P* < .05; ^∗∗∗^*P* < .001; *P* values refer to the differences between clinical subgroups [MCI or AD] and controls).

**Table 2:** Plasma levels of the most important metabolites and metabolite ratios altered in mild cognitive impairment (MCI) and Alzheimer (AD) patients

Metabolites and metabolite ratios	Plasma levels			*P* values			ROC (AUC)	
CO	MCI	AD	CO versus MCI	CO versus AD	MCI versus AD	MCI	AD
Metabolites								
lysoPC a C18:1	23 ± 6	27 ± 8	28 ± 8	NS	.002	NS	—	0.71
lysoPC a C18:2	28 ± 9	38 ± 15	34 ± 10	.0005	NS	NS	0.78	—
PC aa C34:4	2.5 ± 0.6	2 ± 0.4	1.9 ± 0.6	.0005	.00005∗	NS	0.73	0.76
PC aa C36:6	1.3 ± 0.4	1 ± 0.3	1 ± 0.4	.0007	.0008	NS	0.7	0.71
PC aa C38:3	56 ± 13	44 ± 10	51 ± 11	.00005∗	NS	NS	0.78	—
PC aa C40:5	13 ± 3	10 ± 2	11 ± 2	.00002∗	.003	NS	0.77	0.67
PC aa C40:6	33 ± 12	25 ± 6	28 ± 10	.002	NS	NS	0.7	—
Metabolite ratios								
PC aa C34:4/lysoPC a C18:1	0.11 ± 0.04	0.08 ± 0.03	0.07 ± 0.03	.00003	.0000002	NS	0.8	0.83
PC aa C34:4/lysoPC a C18:2	0.10 ± 0.04	0.06 ± 0.02	0.06 ± 0.02	.0000007	.0000009	NS	0.85	0.82
PC aa C36:5/lysoPC a C18:2	1.4 ± 0.8	0.9 ± 0.5	0.9 ± 0.4	.0005†	.0002†	NS	0.73	0.74
PC aa C36:6/lysoPC a C18:1	0.06 ± 0.03	0.04 ± 0.02	0.04 ± 0.01	.00004	.000001	NS	0.75	0.8
PC aa C36:6/lysoPC a C18:2	0.05 ± 0.02	0.03 ± 0.02	0.03 ± 0.01	.000001	.000003	NS	0.83	0.79

Abbreviations: CO, control; MCI, mild cognitive impairment; AD, Alzheimer's disease; ROC, receiver operating characteristic; AUC, area under the ROC curve; SD, standard deviation; NS, not significant; ANOVA, analysis of variance; LSD, least-significant difference.

NOTE. Values are given as mean ± SD (in μM for metabolites). The number of patients was 35 (controls), 33 (MCI), and 43 (AD). Samples were statistically analyzed using ANOVA and Fisher's LSD post hoc test as well as the ROC curve. The *P* values and area under the ROC curve (AUC) are given. For all other parameters, significance was retained after adjustment for multiple testing using the Bonferroni method.

∗ For these markers, significance was retained after adjustment for multiple testing by means of the Bonferroni method: *P* < .05/183 = .0002732. For all other parameters, significance was not retained after adjustment for multiple testing using the Bonferroni method.

† For these markers, significance was “not” retained after adjustment for multiple testing by means of the Bonferroni method, i.e., *P* > .0002732.

**Table 3:** Prediction of MCI and dementia by the ratio between PC aa C34:4 and lysoPC a C18:2: AUC of ROC curve, optimal cutoff value, sensitivity, specificity, positive, and negative predictive value

Measure	MCI versus control		AD versus control	
Estimate	95% CI	Estimate	95% CI
Area under ROC curve (unbiased estimate)∗	0.853	0.743–0.926	0.823	0.717–0.898
Optimal cutoff value for the ratio	0.064†	0.052–0.076	0.0715‡	0.060–0.085
Sensitivity (bias-corrected)§	0.813	0.636–0.928	0.744	0.588–0.865
Specificity (bias-corrected)§	0.857	0.702–0.940	0.771	0.599–0.895
Positive predictive value (bias-corrected)§	0.839	0.667–0.934	0.800	0.650–0.898
Negative predictive value (bias-corrected)§	0.833	0.677–0.925	0.711	0.551–0.831

Abbreviations: MCI, mild cognitive impairment; AUC, area under the ROC curve; ROC, receiver operating characteristic; AD, Alzheimer's disease; CI, confidence interval.

∗ As all subjects included had verified disease status, AUC estimation was unbiased.

† A subject was classified as MCI if the ratio between PC aa C34:4 and lysoPC a C18:2 was <0.064, otherwise as cognitively intact.

‡ A subject was classified as AD if the ratio between PC aa C34:4 and lysoPC a C18:2 was <0.0715, otherwise as cognitively intact.

§ Bias-corrected estimates were derived by cross-validation (leave-one-out method).

**Table 4:** Stability of the lipid metabolites

Healthy controls, Alzheimer patients, and stability	PC aa C34:4	lysoPC a C18:2	Ratio
A			
Day 0	100 ± 1	100 ± 1	100 ± 1
Day 1	91 ± 2 NS	125 ± 2^∗^	80 ± 1^∗∗∗^
Day 2	87 ± 3^∗∗^	136 ± 4^∗∗∗^	70 ± 4^∗∗∗^
Day 3	83 ± 1^∗∗∗^	145 ± 4^∗∗∗^	60 ± 3^∗∗∗^
B			
Day 0	100 ± 5	100 ± 7	100 ± 3
Day 1	80 ± 6^∗^	120 ± 7^∗^	64 ± 6^∗∗∗^
C			
Fresh	100 ± 7	100 ± 6	100 ± 5
2 y—20°C	102 ± 4 NS	105 ± 6 NS	98 ± 5 NS

Abbreviations: NS, not significant; SEM, standard error of the mean; ANOVA, analysis of variance.

NOTE. Stability of plasma lipid metabolites in healthy controls (A) and Alzheimer patients (B): Blood (4 × 2 mL) was collected in EDTA tubes, processed (centrifuged and stored at −80°C) immediately, or after 1–2 or 3 d storage at room temperature. C: Stability at −20°C: Blood was collected, immediately processed, and analyzed the same day, or a plasma aliquot was frozen at −20°C and analyzed after 2 y. Values are given as mean ± SEM % of control. Plasma was analyzed as described in quadruplicate. Statistical analysis was performed by one-way ANOVA with a subsequent Dunnett post hoc test (^∗^*P* < .05; ^∗∗^*P* < .01; ^∗∗∗^*P* < .001).

## Supplementary data

Supplementary Table 1 Quantitative results of all measured metabolites for 43 AD, 33 MCI and 35 control plasma samples obtained with the AbsoluteIDQ® p180 Kit (BIOCRATESLife Science AG, Innsbruck, Austria). Limit of detection (LOD), CV (%) and calibration range are indicated for each metabolite.
